# The B-box module of CYLD is responsible for its intermolecular interaction and cytoplasmic localization

**DOI:** 10.18632/oncotarget.15142

**Published:** 2017-02-07

**Authors:** Songbo Xie, Miao Chen, Siqi Gao, Tao Zhong, Peng Zhou, Dengwen Li, Jun Zhou, Jinmin Gao, Min Liu

**Affiliations:** ^1^ Institute of Biomedical Sciences, College of Life Sciences, Key Laboratory of Animal Resistance Biology of Shandong Province, Shandong Normal University, Jinan 250014, China; ^2^ State Key Laboratory of Medicinal Chemical Biology, College of Life Sciences, Nankai University, Tianjin 300071, China

**Keywords:** CYLD, intermolecular interaction, NF-κB, B-box, deubiquitinase

## Abstract

The tumor suppressor protein cylindromatosis (CYLD), as a microtubule-associated deubiquitinase, plays a pivotal role in a wide range of cellular activities, including innate immunity, cell division, and ciliogenesis. Structural characterization reveals a small zinc-binding B-box inserted within the ubiquitin specific protease (USP) domain of CYLD; however, the exact role for this module remains yet to be elucidated. Here we identify a critical role for the B-box in facilitating the intermolecular interaction and subcellular localization of CYLD. By co-immunoprecipitation assays we uncover that CYLD has the ability to form an intermolecular complex. Native gel electrophoresis analysis and pull down assays show that the USP domain of CYLD is essential for its intermolecular interaction. Further investigation reveals that deletion of the B-box from the USP domain disrupts the intermolecular interaction of CYLD. Importantly, although loss of the B-box has no obvious effect on the deubiquitinase activity of CYLD, it abolishes the USP domain-mediated retention of CYLD in the cytoplasm. Collectively, these data demonstrate an important role for the B-box module of CYLD in mediating its assembly and subcellular distribution, which might be related to the functions of CYLD in various biological processes.

## INTRODUCTION

The tumor suppressor protein cylindromatosis (CYLD) is critically involved in the regulation of diverse signaling pathways, including nuclear factor-κB (NF-κB), transforming growth factor-β (TGF-β), and Wnt/β-catenin, through its deubiquitinase activity towards its substrates [1–4]. It has been well-characterized that CYLD bears three cytoskeletal-associated protein-glycine-conserved (CAP-GLY) domains in the N-terminus, allowing for its binding with microtubules and other proteins, and a ubiquitin specific protease (USP) domain in the C-terminus, which is responsible for its selectivity for Lys63 and Met-1 linked polyubiquitin chains [5–7]. In cells, CYLD engages in multifaceted activities, including cell division, cell migration, angiogenesis, and ciliogenesis [8–12]. Recent studies reveal that in some cases both the deubiquitinase and microtubule binding activity are required, such as in spindle orientation [8], a crucial process for proper cell fate determination, and in G1/S transition [11]. Despite the significance of CYLD in regulating these cellular processes, little is known concerning how CYLD itself is controlled.

The CAP-GLY domains are conserved modules residing in many functionally diverse proteins, including the dynactin complex subunit p150^glued^, cytoplasmic linker protein 170 (CLIP-170), and the kinesin protein KIF13B [13–15]. The presence of CAP-GLY domains allows CYLD to interact with microtubules and other proteins, such as histone deacetylase 6 (HDAC6) [11], a key regulator of microtubule dynamics, and NF-κB-essential modulator (NEMO), a cardinal modulator of NF-κB signaling [16]. CYLD interacts with microtubule via its first two CAP-GLY domains [5]. However, compared to the canonical microtubule binding proteins, the interaction between CYLD and microtubules seems relative weak, as CYLD is predominantly located in the cytoplasm and only slightly colocalization with microtubules is observed in cells [17, 18]. Given the critical involvement of CYLD-mediated microtubule dynamics in different cellular activities, understanding the mechanisms underlying CYLD-microtubule interaction and CYLD subcellular localization is of pivotal importance.

Recent studies reveal that CLIP-170 and end-binding protein 1 (EB1), two important microtubule plus-end tracking proteins (+TIPs), can form dimer to exert their roles in the regulation of microtubule assembly [19, 20]. Interestingly, structural characterization of the CYLD USP domain reveals a small zinc-binding B-box module [21]. However, the exact function of the B-box remains not fully elucidated. In this study, we sought to define the special role of the CYLD B-box module in mediating intermolecular interaction, subcellular localization, and deubiquitinase activity of CYLD.

## RESULTS

### Identification of CYLD intermolecular interaction in cells

**Figure 1:**
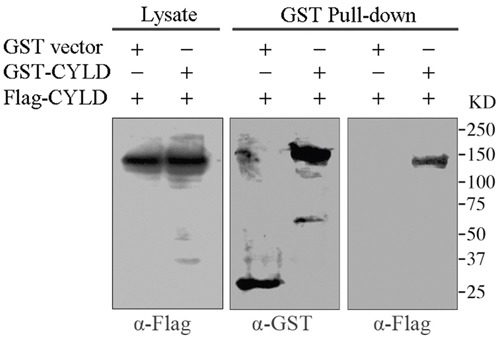
GST pull-down assays to analyze CYLD intermolecular interaction HEK293T cells were co-transfected with GST vector or GST-tagged CYLD plasmid together with Flag-tagged CYLD plasmid. GST or GST-tagged CYLD were pull-downed and the precipitations were determined with anti-GST and anti-Flag antibodies, and cell lysates were analyzed as a control.

In cells, CYLD acts in concert with the +TIPs protein EB1 to regulate microtubule dynamics [22]. Recent study reveals that EB1 can form dimers by an intermolecular interaction, which leads to the autoinhibition of EB1 activity [19, 23]. The dimerization-caused autoinhibition of EB1 prompts us to investigate whether CYLD has similar behavior. To test this hypothesis, we co-transfected GST-tagged CYLD and Flag-tagged CYLD into HEK293T cells and examined whether Flag-tagged CYLD was present in the GST pull-downed precipitation. As shown in Figure 1 (right panel), Flag-tagged CYLD was detected from the pull-downs of GST-tagged CYLD, indicating an intermolecular interaction of CYLD in the cell.

### The USP domain is critical for CYLD intermolecular interaction

**Figure 2:**
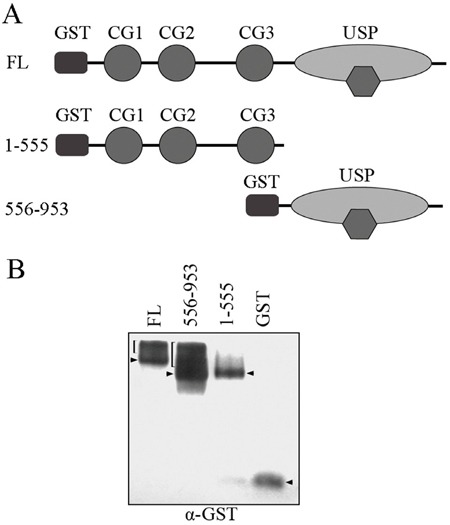
Native PAGE electrophoresis analysis for CYLD complex formation **A.** Schematic illumination of GST-tagged CYLD full-length (FL), CYLD-N (1-555 version), and CYLD-C (556-953 version) fragments. **B.** HEK293T cells were transfected with GST vector or GST-tagged CYLD truncations. Proteins in the cell lysates were separated by native PAGE electrophoresis and were then denatured and transferred to PVDF membrane. Anti-GST antibody was used to detect GST-tagged proteins. Predicted intermolecular complexes are indicated by brackets, arrowheads indicate monomers. Note that the presence of endogenous CYLD can affect the migration patterns.

**Figure 3:**
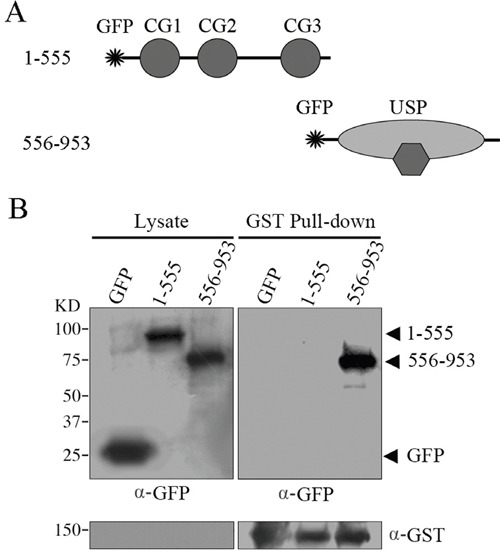
GST pull-down assays to examine the domains responsible for CYLD intermolecular interaction **A.** Schematic illumination of GFP-tagged CYLD-N (1-555 version) and CYLD-C (556-953 version) fragments. **B.** HEK293T cells were co-transfected with GFP vector or GFP-tagged CYLD truncations together with GST-tagged CYLD plasmid. GST-tagged CYLD were pull-downed and the precipitations were determined with anti-GFP and anti-GST antibodies, and cell lysates were analyzed as a control.

Both the CAP-GLY domain and USP domain have been shown to mediate protein-protein interactions. To define the molecular mechanism of which domain is responsible for CYLD intermolecular interaction, we constructed GST-tagged CYLD truncations (Figure 2A) and then carried out native PAGE electrophoresis to analyze the formed complexes. Interestingly, full-length CYLD or the C-terminal truncation (556-953 version) that contains the USP domain was detected to migrate to different positions in the native gel that could suggest formation of complexes. However, CYLD N-terminal truncation (1-555 version) or GST itself did not show multi-band migration, and was likely present as monomers in the cell (Figure 2B). These observations suggest that the USP domain might mediate the CYLD intermolecular interaction. To confirm this hypothesis, we constructed GFP-tagged CYLD truncations (Figure 3A) and co-transfected cells with GST-tagged CYLD together with GFP-tagged CYLD-N-terminal truncation (1-555 version) or GFP-tagged CYLD-C-terminal truncation (556-953 version), and examined the pull-downed precipitations with anti-GFP antibodies. As shown in Figure 3B, GFP-tagged CYLD-C-terminal truncation, but not the N-terminal truncation, was detected in the precipitation, confirming that the USP domain is required for CYLD intermolecular interaction.

### The B-box module mediates CYLD intermolecular interaction

**Figure 4:**
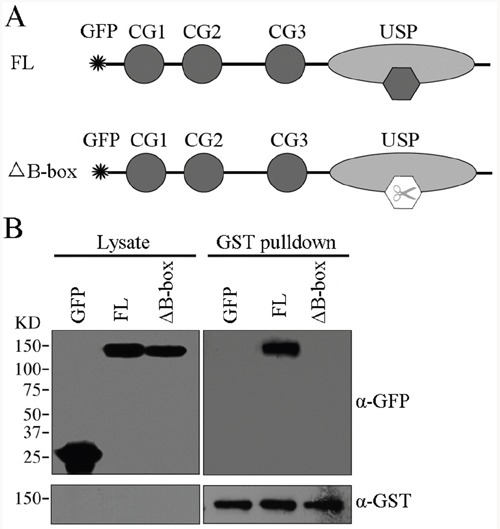
The B-box module is required for CYLD intermolecular interaction **A.** Schematic illumination of GFP-tagged CYLD full-length (FL) and GFP-tagged CYLD^ΔB-box^ (Δ786-837). **B.** HEK293T cells were co-transfected with GST-tagged CYLD plasmid together with GFP-tagged CYLD or GFP-tagged CYLD^ΔB-box^ plasmids. GST-tagged CYLD were pull-downed and the precipitations were determined with anti-GFP and anti-GST antibodies, and cell lysates were analyzed as a control.

Several studies suggest that the small zinc-binding B-box module, a conserved motif residing in eukaryotic cells, is responsible for protein-protein interaction [24, 25]. We thus investigated whether the B-box module within the USP domain is involved in CYLD intermolecular interaction. We transfected GST-tagged CYLD together with GFP-CYLD or GFP-CYLD^ΔB-box^ (^Δ^786-837) plasmids (Figure 4A) into the HEK293T cells and GST pull-down was performed to test the requirement of B-box module for CYLD intermolecular interaction. Indeed, deletion of the B-box module abolished the intermolecular interaction of CYLD, as CYLD without the B-box module (CYLD^ΔB-box^) could not be detected in the precipitations (Figure 4B). In conclusion, our data suggest that CYLD forms homopolymer through interactions mediated by the B-box module located in the USP domain.

### The B-box module is required for CYLD cytoplasmic localization

**Figure 5:**
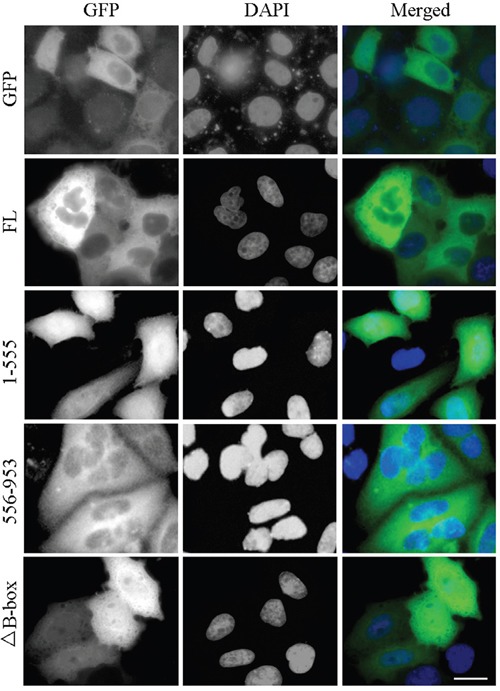
The B-box module is required for proper localization of CYLD in the cell HEK293T cells were transfected with GFP-tagged CYLD or its various truncations. Fixed cells were stained with DAPI to visualize nuclei. Scale bar: 20 μm.

Next we analyzed the impact of the B-box module on the biological functions of CYLD. We first examined its effect on CYLD subcellular distribution. Cells were transfected with GFP-tagged CYLD or its various truncations. Wild-type full-length CYLD localized in the cytoplasm (Figure 5). Loss of the USP domain from CYLD (1-555 version) led to an increase in nuclear localization, while the CYLD USP truncation (556-953 version) itself was retained in the cytoplasm (Figure 5). Moreover, deletion of the B-box from CYLD (CYLDΔ^B-box^) disrupted the retention of CYLD in the cytoplasm (Figure 5). These data suggest the B-box module in the USP domain is critical for CYLD subcellular distribution and thus might regulate various biological functions of CYLD.

### The B-box module is dispensable for its deubiquitinase activity

**Figure 6:**
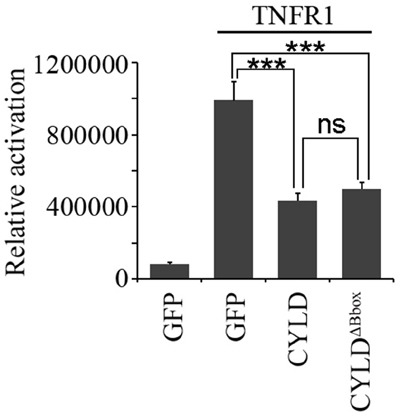
The B-box module is dispensable for the NF-κB suppressing activity of CYLD HEK293T cells were transfected with pGL4.32-NF-κB-Luc and pcDNA3-LacZ plasmids together with or without pcDNA3-Flag-TNFR1 plasmid in the presence of pEGFPC1-CYLD or pEGFPC1-GFP-CYLD^ΔB-box^ plasmids. Luciferase and β-galactosidase activities were examined. *** indicates P<0.001; ns, not significant. Error bars indicate SEM.

Additionally, we investigated the requirement of the B-box module for the deubiquitinase activity of CYLD. As CYLD is a crucial negative regulator of NF-κB signaling, we thus examined the effect of the B-box module on NF-κB-mediated luciferase reporter activity. Cells transfected with tumor necrosis factor receptor 1 (TNFR1) had an elevated NF-κB-mediated reporter activity and overexpression of CYLD significantly suppressed the TNFR1-induced NF-κB activation (Figure 6). Interestingly, overexpression of CYLD^ΔB-box^ could also efficiently suppress the TNFR1-induced NF-κB activation (Figure 6), suggesting that the B-box module is dispensable for the CYLD deubiquitinase activity. A functional USP domain in CYLD^ΔB-box^ protein also suggests that deletion of the B-box module does not disrupt the folding of this domain. In summary, the B-box module of CYLD is critical for the intermolecular interaction and subcellular localization, but not deubiquitinase activity.

## DISCUSSION

CYLD is an important deubiquitinase and microtubule-binding protein that involves multiple cellular activities, including cell cycle progression, innate immune responses, and cancer. Although the conserved CAP-GLY domains provide structural basis for the interactions of CYLD with microtubules and many other proteins, an interesting finding is that EB1 containing a known CAP-GLY domain-interacting motif interacts with the C-terminal USP domain of CYLD other than the CAP-GLY domains, implicating a previously unknown role for the USP domain in mediating protein-protein interactions. In this study, we find that the USP domain is critical for CYLD intermolecular interaction. Consistently, other studies reveal CYLD interacts with SPATA2 via the USP domain, supporting the notion that USP domain might also be a critical element for protein-protein interactions [26, 27].

**Figure 7:**
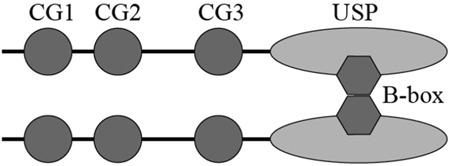
Schematic illustration of proposed CYLD dimerization via the B-box module

The B-box module is a conserved domain primarily existing in tripartite motif (TRIM) family proteins. Several studies suggest B-box module may act as a protein-interacting motif to mediate the recognition of TRIM proteins with their substrates [24, 28]. In addition, the role of B-box in promoting protein self-association and forming dimers has been reported [29]. The TRIM5 B-box/B-box interactions lead to the formation of trimmers and subsequently a hexagonal net, allowing for TRIM5 to effectively assemble and recognize the HIV-1 capsids [25]. The CYLD B-box module inserted within the USP domain is newly identified but its function remains yet to be characterized [21]. In the present study, we reveal that the CYLD B-box is responsible for the intermolecular interaction. The observation that deletion of the B-box module (CYLD^ΔB-box^) completely abolishes its interaction with wild-type full-length CYLD (Figure 4), suggests that CYLD intermolecular interaction is also mediated by B-box/B-box interaction. Given the small size of the B-box module (52 amino acids) compared to the whole USP domain (398 amino acids), dimerization of CYLD might block further polymerization of monomer CYLD. We thus hypothesize that CYLD forms homodimers in the cell (Figure 7), which is in line with the findings recently unraveled by Elliott et al. [30].

Our further study demonstrates an essential role for the B-box module in retaining CYLD in the cytoplasm, which is consistent with observations reported by Komander et al. [21]. However, the molecular mechanism by which B-box regulates CYLD cytoplasmic retention remains unclear. One possibility is that B-box-mediated CYLD intermolecular interaction stimulates its binding with other cytosol proteins, since a large number of CYLD-interacting partners have been reported [31]. In supporting of this notion, the CYLD intermolecular interaction promotes its recruitment to the linear ubiquitin chain assembly complex (LUBAC) and TNFR1 complex [26, 27, 30]. In addition, given that the CYLD intermolecular interaction can provide additional GAP-GLY domains to crosslink microtubules, it is possible that the intermolecular interaction enhances the CYLD-microtubule binding, and thereby leads to CYLD cytoplasmic localization. These hypotheses open future directions to investigate whether and how CYLD intermolecular interaction affects the microtubule dynamics and microtubule-related cellular processes.

## MATERIALS AND METHODS

### Antibodies, chemicals, and plasmids

Antibodies against GFP, Flag, and GST were purchased from Sigma-Aldrich, and horseradish peroxidase-conjugated secondary antibodies were obtained from Amersham Biosciences. Glutathione (GSH) agarose beads and dual-Luciferase Reporter Assay System were from Promega. The mammalian expression plasmid for GST-CYLD, GST-CYLD (1-555), and GST-CYLD (556-953) were constructed by inserting the indicated cDNA into the pEBG vector. The mammalian expression plasmid for Flag-CYLD was obtained as previously described [5]. The mammalian expression plasmid for GFP-CYLD, GFP-CYLD (1-555), GFP-CYLD (556-953), and GFP-CYLD^ΔB-box^ (Δ786-837) were generated by cloning the indicated cDNA into the pEGFPC1 vector.

### Cell culture and transfection

HEK293T cells were cultured in the DMEM medium supplemented with 10% fetal bovine serum at 37°C in a humidified atmosphere with 5% CO_2_. Plasmids were transfected or co-transfected into cells with Lipofectamine 2000 (Invitrogen).

### Immunoblot analysis and GST pull-down assays

For immunoblot analysis, proteins were separated by SDS-PAGE and transferred onto polyvinylidenedifluoride membranes (Millipore). Then the membranes were blocked with 5% fat-free milk, and probed sequentially with primary antibodies and horseradish peroxidase-conjugated secondary antibodies. Target proteins were visualized with enhanced chemiluminescence detection reagent (Pierce Biotechnology). For GST pull-down, cells transfected with GST-tagged plasmids were lyzed and cell lysates were incubated with glutathione-sepharose 4B beads at 4 °C overnight. The pulled-down proteins were then visualized by immunoblotting.

### Native PAGE electrophoresis

Cell lysates were mixed with sample buffer (62.5 mM Tris-HCl, pH 6.8, 25% glycerol, 1% Bromophenol Blue) and loaded into the 10% native PAGE gel. The electrophoresis was carried out at 4 °C with a set of 15 voltage. Proteins in the gel were denatured with transfer buffer containing 2% SDS and were then transferred onto polyvinylidenedifluoride membranes and analyzed by immunoblotting.

### Immunofluorescence microscopy

Cells grown on glass coverslips were transfected with GFP-tagged plasmids for 48 hours. Then cells were fixed with 4% paraformaldehyde at room temperature for 20 minutes. nuclei were stained with DAPI for 5 minutes. Coverslips were mounted with 90% glycerol in PBS and then examined with an Axio Observer A1 fluorescence microscope (Carl Zeiss, Inc.).

### Luciferase reporter assay

293T cells were co-transfected with the NF-κB luciferase reporter plasmid pGL4.32-NF-κB-Luc, β-galactosidase-expressing plasmid pcDNA3-LacZ, pcDNA3-Flag-TNFR1 plasmid, together with pEGFPC1-CYLD or pEGFPC1- GFP-CYLD^ΔB-box^ plasmid. 48 hour later, cells were lyzed and the luciferase activity was measured using the FB12 luminometer (Berthold Detection Systems) and normalized to β-galactosidase activity.

### Statistics

All the experiments were independently performed for three times, and analysis of statistical significance was determined by the ANOVA test for multiple comparisons.
